# Combinational Treatment of Curcumin and Quercetin against Gastric Cancer MGC-803 Cells *in Vitro*

**DOI:** 10.3390/molecules200611524

**Published:** 2015-06-22

**Authors:** Jian-Ye Zhang, Min-Ting Lin, Meng-Jia Zhou, Tao Yi, Yi-Na Tang, Si-Li Tang, Zhi-Jun Yang, Zhong-Zhen Zhao, Hu-Biao Chen

**Affiliations:** 1 School of Chinese Medicine, Hong Kong Baptist University, Kowloon Tong, Kowloon, Hong Kong, China; E-Mails: jianyez@163.com (J.-Y.Z.); aknet5jia@hotmail.com (M.-J.Z.); yitao@hkbu.edu.hk (T.Y.); 11467312@life.hkbu.edu.hk (Y.-N.T.); yzhijun@hkbu.edu.hk (Z.-J.Y.); zzzhao@hkbu.edu.hk (Z.-Z.Z.); 2 School of Pharmaceutical Sciences, Guangzhou Medical University, 195 Dongfeng Road West, Guangzhou 510182, China; E-Mails: minting113@sina.com (M.-T.L.); tangsilisixin@126.com (S.-L.T.)

**Keywords:** combined effect, quercetin, curcumin, apoptosis, AKT, ERK

## Abstract

Gastric cancer remains a major health problem worldwide. Natural products, with stronger antitumor activity and fewer side effects, are potential candidates for pharmaceutical development as anticancer agents. In this study, quercetin and curcumin were chosen for testing and were applied separately and in combination to human gastric cancer MGC-803 cells. The MTT assay was used to evaluate cell growth inhibition. Annexin V-FITC/PI was carried out to measure apoptosis rate. Flow cytometry was performed to analyze mitochondrial membrane potential levels. Western blots were applied to detect expression of cytochrome c, total and phosphorylated ERK and AKT. Combined treatment with curcumin and quercetin resulted in significant inhibition of cell proliferation, accompanied by loss of mitochondrial membrane potential (ΔΨm), release of cytochrome c and decreased phosphorylation of AKT and ERK. These results indicate that the combination of curcumin and quercetin induces apoptosis through the mitochondrial pathway. Notably, effect of combined treatment with curcumin and quercetin on gastric cancer MGC-803 cells is stronger than that of individual treatment, indicating that curcumin and quercetin combinations have potential as anti-gastric cancer drugs for further development.

## 1. Introduction

Gastric cancer remains a leading cause of cancer deaths worldwide. Although mortality rates of gastric cancer have declined over the past few decades, the disease still has a poor prognosis and remains a major health problem [1], as many patients present advanced or metastatic disease at the time of diagnosis. Given the relatively short overall survival of advanced gastric cancer patients and the palliative nature of systemic chemotherapy, chemotherapeutic agents should be selected based on efficacy, low toxicity, and convenient administration [2]. 

**Figure 1:**
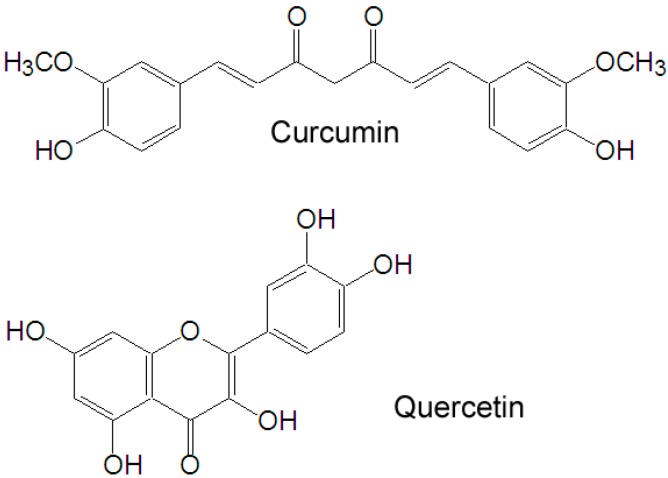
Chemical structures of quercetin and curcumin.

Natural products, with stronger antitumor activity and fewer side effects, have gained increasing scientific attention in recent years. Curcumin (Figure 1), a yellow polyphenol, is an active principle of the perennial herb *Curcuma longa* (commonly known as turmeric). It has been shown to suppress multiple signaling pathways and inhibit cancer cell proliferation, invasion, metastasis and angiogenesis [3]. The clinical potential of curcumin, however, has been limited due to its low bioavailability (<2%) [4,5]. To overcome the limitation, modern techniques such as structural modifications and various formulations have been explored. Quercetin (Figure 1) is a plant-derived flavonoid which possesses antioxidant, anti-inflammatory and anticancer activities, and which is a dual inhibitor of cytochrome P450 3A4 and modulator of ABCB1. Due to these activities, when combined with other drugs, quercetin can enhance the bioavailability of these drugs [6]. In particular, when delivered together with curcumin, quercetin was found to increase the uptake of curcumin into human colon carcinoma WiDr cells and improve curcumin bioavailability in a self-microemulsifying drug delivery system [7,8]. Herein, combination of curcumin and quercetin is believed to improve the bioavailability and clinical potential of curcumin. Nevertheless, the effect of curcumin and quercetin in combination on human gastric cancer has not been reported. In this paper, we reported studies of the combined effect of curcumin and quercetin on gastric cancer MGC-803 cells and of the mechanism by which this effect occurs.

## 2. Results and Discussion

The cytotoxicity of curcumin and quercetin individually and in combination was determined by the MTT assay to evaluate their antitumor activity. It was shown that the combination of curcumin and quercetin exhibited stronger anticancer activity than individual treatment. The IC_50_ values of curcumin and quercetin against MGC-803 cells were 9.32 ± 1.06 μM and 23.35 ± 2.14 μM. Growth inhibitory rates were 12.26% ± 3.09%, 40.98% ± 4.33%, 51.44% ± 3.90%, 76.99% ± 3.06% and 84.37% ± 4.99% after cells were treated with 10.0 μM quercetin, 5.0 μM curcumin, 10.0 μM curcumin, 10.0 μM quercetin + 5.0 μM curcumin and 10.0 μM quercetin + 10.0 μM curcumin, respectively.

Curcumin exhibits anti-cancer activities both *in vitro* and *in vivo* through a variety of mechanisms. It inhibits proliferation and induces apoptosis in a wide array of cancer cell types *in vitro*, including cells from cancers of the bladder, breast, lung, pancreas, prostate, cervix, head and neck, ovary, kidney, brain, bone marrow, and skin [9]. In former study, we found that quercetin was effective in both sensitive cancer cells and ABCB1-induced MDR cancer cells and induced apoptosis via mitochondrial pathway [10].

**Figure 2:**
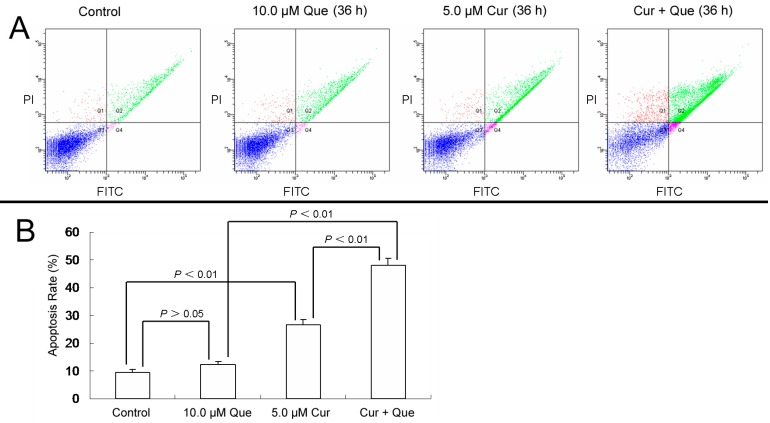
Combination of quercetin and curcumin significantly triggered apoptosis of MGC-803 cells. MGC-803 cells were treated with quercetin (10.0 µM), curcumin (5.0 µM) or a combination of the two for 36 h. Apoptosis rates were then measured by flow cytometry. (**A**) Apoptosis induced by quercetin and/or curcumin in MGC-803 cells. (**B**) Data analysis of (**A**). Values are expressed as mean ± SD of three determinations. *p* < 0.05 and *p* < 0.01 represent a significant difference among groups. Que and Cur are abbreviations of quercetin and curcumin, respectively.

Defects in apoptosis are critical in tumorigenesis and resistance to therapy [11]. To clarify whether combination of curcumin and quercetin induced cell apoptosis in MGC-803 cells, Annexin-V and PI double staining were performed. Apoptotic rates were 9.4% ± 1.4%, 12.3% ± 1.2%, 26.5% ± 2.1% and 47.1% ± 2.4% for control, 10.0 μM quercetin, 5.0 μM curcumin and 10.0 μM quercetin + 5.0 μM curcumin, respectively (Figure 2). Cell apoptosis increased significantly when cells were treated with the combination of quercetin and curcumin.

Apoptotic cell death can be induced through the extrinsic (also called receptor-mediated) or the intrinsic (also called mitochondria-mediated) signalling pathways. Within the mitochondrial pathway of apoptosis, chemotherapeutic drugs or other stimuli change the properties of the inner mitochondrial membrane and subsequently cause the loss of mitochondrial membrane potential (ΔΨm) and release of cytochrome c from the mitochondria into the cytosol. Collapse of ΔΨm and release of cytochrome c are biochemical markers in apoptosis through the mitochondrial pathway [12]. To elucidate the mechanism of curcumin and quercetin-induced apoptosis, biochemical markers in the mitochondrial apoptotic pathway were examined.

**Figure 3:**
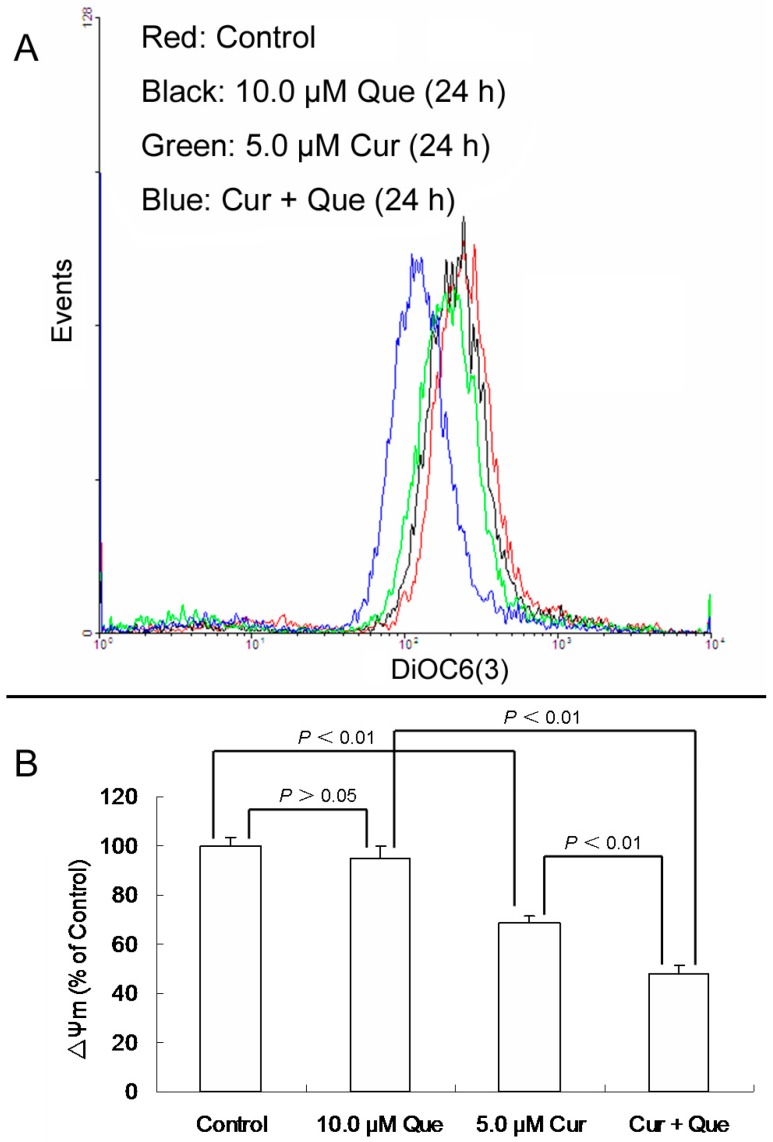
Effect of quercetin and curcumin on ΔΨm in MGC-803 cells. After treatment with quercetin (10.0 µM) and/or curcumin (5.0 µM) for 24 h, cells were incubated with DiOC6(3) and then measured by flow cytometry. (**A**) Collapse of ΔΨm was observed in MGC-803 cells. (**B**) ΔΨm levels are expressed as percentage of MFI compared to control group. Data are expressed as mean ± SD of at least three determinations. *p* < 0.05 and *p* < 0.01 represent a significant difference among groups.

After MGC-803 cells were exposed to 10.0 μM quercetin and/or 5.0 μM curcumin for 24 h, ΔΨm for 10.0 μM quercetin, 5.0 μM curcumin and combination group were 94.66 ± 5.09% (*p* > 0.05), 68.49% ± 3.18% (*p* < 0.01) and 48.14% ± 3.08% (*p* < 0.01) of the control, respectively (Figure 3). Quercetin alone didn’t change the ΔΨm level, while curcumin alone or in combination with quercetin significantly decreased the ΔΨm level. Moreover, the effect of the combination was stronger than that of curcumin alone, which was consistent with the result of apoptosis detection.

**Figure 4:**
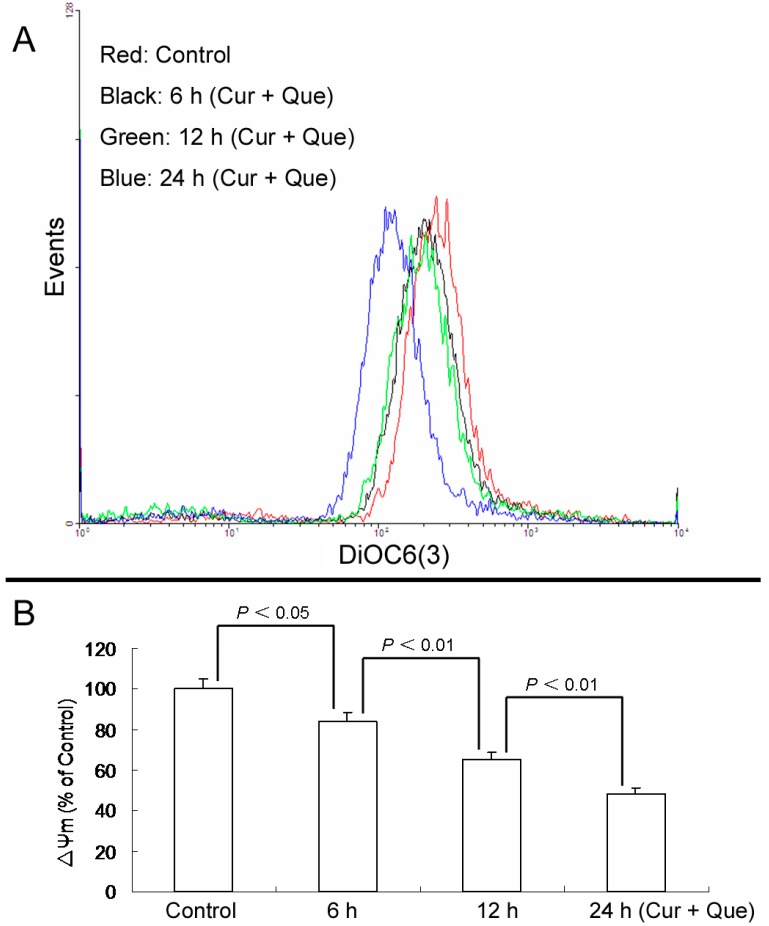
Combination of quercetin and curcumin decreased ΔΨm in a time-dependent manner. After treatment with combination of quercetin and curcumin for 6, 12 and 24 h, cells were incubated with DiOC6(3) and then measured by flow cytometry. (**A**) Collapse of ΔΨm was observed in MGC-803 cells. (**B**) ΔΨm levels are expressed as percentage of MFI compared to control group. Data are expressed as mean ± SD of at least three determinations. *p* < 0.05 and *p* < 0.01 represent a significant difference among groups.

Furthermore, loss of ΔΨm induced by a combination of 10.0 μM quercetin and 5.0 μM curcumin was time-dependent. After cells were exposed to a combination of 10.0 μM quercetin and 5.0 μM curcumin for 6, 12 and 24 h, ΔΨm were 84.10% ± 4.39%, 65.41% ± 3.18% and 48.14% ± 3.39% of control, respectively (Figure 4).

**Figure 5:**
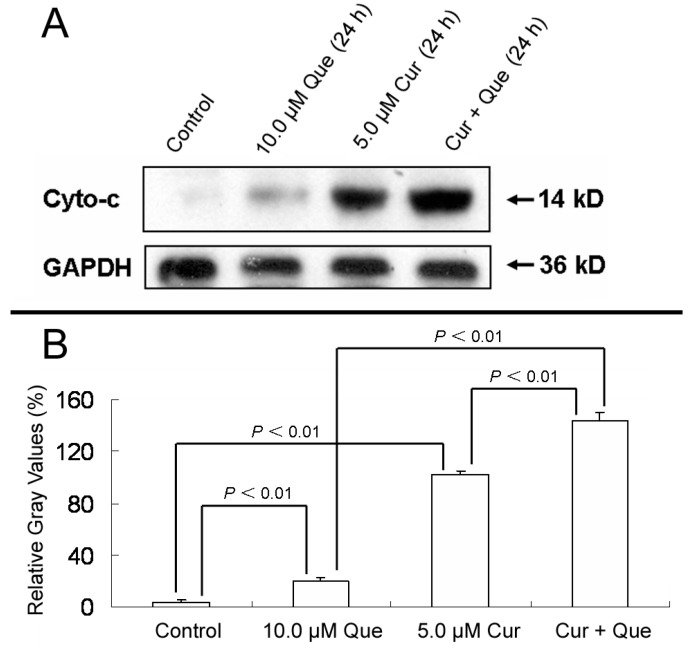
Effect of quercetin and curcumin on cytochrome c release. MGC-803 cells were treated with quercetin (10.0 µM), curcumin (5.0 µM) or a combination of the two for 24 h. The protein expression of cytochrome c was then measured by western blot. (**A**) Expression of cytochrome c in MGC-803 cells. (**B**) Gray intensity analysis of western blot results of (**A**). GAPDH (36 kDa) was used to ensure equal protein loading. Data are expressed as mean ± SD of at least three determinations. *p* < 0.05 and *p* < 0.01 represent a significant difference among groups.

In addition, after MGC-803 cells were treated with 10.0 μM quercetin and/or 5.0 μM curcumin for 24 h, the ratios of densitometric values (cytochrosome c/GAPDH) for control, 10.0 μM quercetin, 5.0 μM curcumin and combination group were 3.94% ± 1.50%, 20.16% ± 2.65%, 102.10% ± 2.73% and 144.09% ± 5.62%, respectively (Figure 5). The results depicted in Figure 3, Figure 4 and Figure 5 were in good agreement with Annexin V-FITC/PI apoptosis detection, indicating that the combination of quercetin and curcumin triggered apoptosis via the mitochondrial pathway. Moreover, the effect of combined treatment was stronger than that of individual treatment.

**Figure 6:**
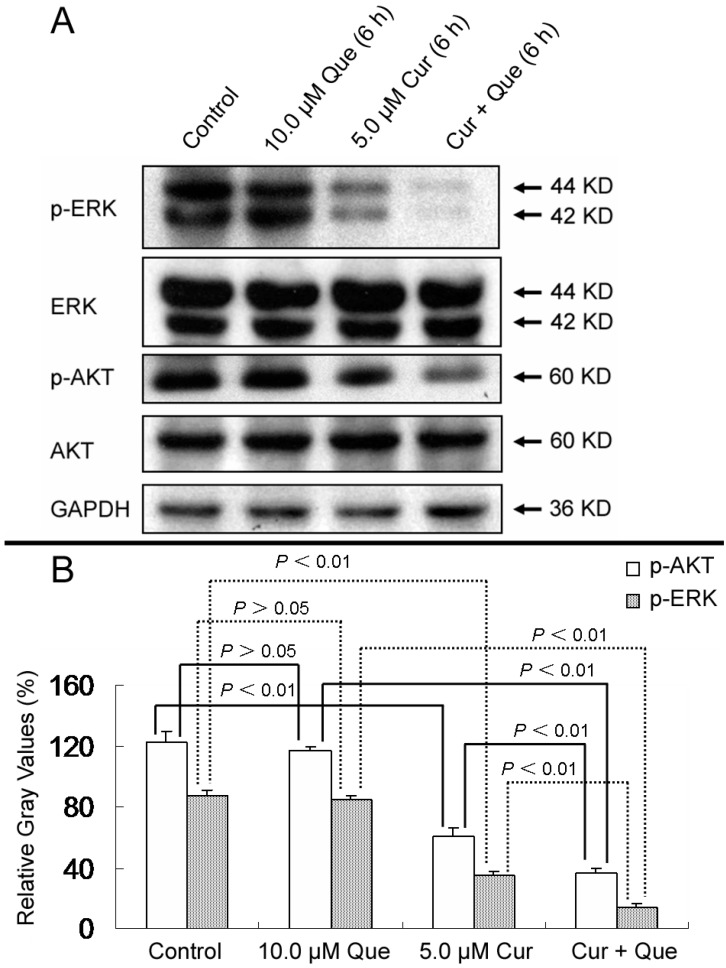
Effect of quercetin and curcumin on the expression of total and phosphorylation forms of AKT and ERK. MGC-803 cells were treated with quercetin (10.0 µM), curcumin (5.0 µM) or a combination of the two for 6 h; the protein expressions of p-ERK, ERK, p-AKT and AKT were then measured by western blot. (**A**) Expression of p-ERK, ERK, p-AKT and AKT in MGC-803 cells. (**B**) Gray intensity analysis of western blot results of p-AKT and p-ERK in (A). GAPDH (36 kDa) was used to ensure equal protein loading. Data are expressed as mean ± SD of at least three determinations. *p* < 0.05 and *p* < 0.01 represent a significant difference among groups.

Given that cancer is a multi-step process, antitumor activities generally involve multiple pathways. For example, curcumin can modulate various molecular targets including transcription factors, growth factors and their receptors, cytokines, enzymes, and genes regulating cell proliferation and apoptosis [13]. Deregulation of MAPK and PI3K pathways play important roles in cell proliferation, survival, migration, differentiation and metabolism. Blockade of these two pathways with combinations of signaling inhibitors might result in a more efficient anti-tumor effect, as compared with treatment with a single agent [14]. AKT and ERK, which are activated by phosphorylation, are novel molecules in the MAPK and PI3K pathways. As shown in Figure 6, phosphorylations of AKT and ERK were dramatically decreased by treatment with the combination of quercetin and curcumin compared with curcumin alone. Treatment with quercetin showed no effect on AKT and ERK phosphorylation (*p* > 0.05). Densitometric ratios (%) of p-ERK/ERK for control, 10.0 μM quercetin, 5.0 μM curcumin and combination group were 87.01% ± 4.39%, 84.25% ± 3.32%, 34.89% ± 3.24% and 14.14% ± 2.18%, respectively. Densitometric ratios (%) of p-AKT/AKT for those were 122.04% ± 7.23%, 116.56% ± 3.09%, 60.98% ± 5.61% and 36.49% ± 2.85%, respectively. All treatments had no effect on total proteins levels. These results further indicate that the MAPK and PI3K pathways are also responsible for quercetin and curcumin-induced cell growth inhibition. To support further development of these two phytochemicals, investigation about the combined effect of quercetin and curcumin on MAPK, PI3K and other pathways is still in need.

**Figure 7:**
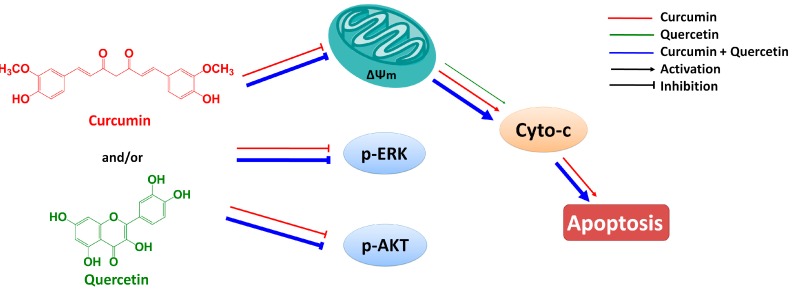
Combined effect of quercetin and curcumin on gastric cancer MGC803 cells.

In summary, curcumin has low aqueous solubility and undergoes extensive first pass metabolism following oral dosing. Early attempts to develop curcumin as an anticancer agent were frustrated largely by its low bioavailability. In recent years, some research fellows have found that quercetin could enhance the bioavailability of curcumin by increasing its uptake into human carcinoma cells [4,5,6,7,8]. Thus, it is possible for curcumin in combination with quercetin to take effect *in vivo*. In this paper, we reported studies of the combined effect of curcumin and quercetin on gastric cancer MGC803 cells and of the mechanism by which this effect occurs. Results show that the combination of curcumin and quercetin can inhibit the phosphorylations of AKT and ERK and induce apoptosis via mitochondrial pathway (Figure 7). Furthermore, effect of combined treatment with curcumin and quercetin is stronger than that of individual treatment. Combination of curcumin and quercetin has the potential as anti-gastric cancer drug for further development.

## 3. Experimental Section

### 3.1. Chemicals and Antibodies

Quercetin and curcumin of 98% purity were purchased from Tianjin Shilan Technology Co. Ltd (Tianjin, China). 3,3ʹ-Dihexyloxacarbocyanine iodide (DiOC6(3)) and 3-(4,5-dimethyl-2-thiazolyl)2,5-diphenyl-2H-tetrazolium bromide (MTT) were purchased from Sigma Chemical Co. (St. Louis, MO, USA). Annexin V-FITC/PI Apoptosis Detection Kit was purchased from KeyGEN Biotech (Nanjing, China). Primary antibodies against cytochrome *c*, AKT, p-AKT, ERK, p-ERK and GAPDH were obtained from Signalway Antibody Co. (College Park, MD, USA) or Santa Cruz Co. (Dallas, TX, USA). Secondary anti-mouse and anti-rabbit IgG-HRP were products of KangChen Biotechnology Co. (Shanghai, China). All culture media and growth supplements were acquired from Gibco-BRL Co. (Gaithersburg, MD, USA). Other routine reagents used were of analytical or HPLC grade and purchased from local commercial sources.

### 3.2. Cell Culture

MGC-803 cells (human gastric cancer cell line) were cultivated in RPMI 1640 medium containing 100 U/mL penicillin, 100 μg/mL streptomycin and 10% FBS in an incubator with a humidified atmosphere of 5% CO_2_ at 37 °C.

### 3.3. Cell Viability Assay

MGC-803 cells were plated in 96-well plates and allowed to adhere 24 h. Cells were then incubated with varying doses of indicated compounds (quercetin, curcumin, combination of quercetin and curcumin, DMSO). After 3 days, MTT was added to each well and plates were incubated for 4 h. Formazan crystals were dissolved with 200 μL DMSO and absorbance at 540 nm was measured by microplate reader with 655 nm as reference filter. Cell survival was calculated as: survival (%) = (mean experimental absorbance/mean control absorbance) × 100% [15].

### 3.4. Apoptosis Detection

Apoptosis rate was detected by the Annexin V-FITC/PI Apoptosis Detection Kit according to the manufacturer’s instruction. Quercetin (10.0 µM) and curcumin (5.0 µM) for individual or combination treatment were cultured with MGC-803 cells for 36 h. Cells were then harvested and resuspended in 0.5 mL binding buffer containing Annexin-V (1:50) and 40 ng/sample of PI for 30 min at 37 °C in the dark. The samples were analyzed by a flow cytometer (BD FASCanto) and CellQuest software. Apoptosis rate (%) = (the number of apoptotic cells/the number of total cells observed) × 100% [16].

### 3.5. Mitochondrial Membrane Potential (Δψm) Measurement

ΔΨm levels were determined by flow cytometry with the mitochondrial tracking fluorescent DiOC6(3) as previously described [17]. MGC-803 cells were plated to a culture dish for 24 h and exposed to quercetin and/or curcumin for indicated time. Samples were stained with 40 nM DiOC6(3) at 37 °C for 20 min in the dark, and measured by BD FASCanto Flow Cytometer (excitation/emission at 484/501 nm). At least 10,000 cells were determined for each sample. The data obtained from flow cytometry were analyzed by CellQuest software and expressed as MFI. The expressed data were the results of three independent determinations.

### 3.6. Western Blot

As previously described [18], MGC-803 cells during the logarithmic growth phase were seeded in a culture dish at a density of 5 × 10^6^ cells/well. After 24 h incubation, cells were treated with curcumin and/or quercetin for 6–24 h. Then, cells were harvested and washed twice with ice-cold PBS.

For whole cell lylates, Eppendorff tubes (1.5 mL) containing cells was centrifuged at 110 *g* for 5 min to discard the supernatant. The pellet was vortexed, and 100 μL of 1× loading buffer for every well was added. After heating at 100 °C for 20 min, the lysates in the Eppendorff were centrifuged at 15,000 *g* for 10 min and the supernatant was collected.

For the subcellular fraction, cells were suspended in Eppendorff tubes (1.5 mL) with a 5-fold volume of ice-cold cell extract buffer and incubated for 40 min at 4 °C. Then, the cells were centrifuged at 110 *g* for 10 min at 4 °C. The supernatant was subsequently centrifuged at 15,000 *g* for 15 min at 4 °C, and the final supernatant was used as the cytosolic fraction. Then, 5 × loading buffer was added to the above obtained supernatant, and the mixture was boiled at 100 °C for 15 min.

Proteins were then separated on 8%–12% sodium SDS-PAGE, electrotransferred to PVDF membranes (Millipore, Billerica, MA, USA) and probed with protein specific antibodies followed by HRP-conjugated secondary antibody. Protein bands were detected with a PhototopeTM-HRP Detection Kit (Cell Signaling, Danvers, MA, USA) on Kodak medical X-ray processor (Kodak, Rochester, NY, USA). The cytochrome c, p-AKT, AKT, p-ERK and ERK protein were detected by specific primary antibody in the ratio of 1:1,000. HRP-conjugated secondary antibodies were diluted in the ratio of 1:2000.

### 3.7. Statistical Analysis

All quantitative values are expressed as mean values ± SD of at least three independent experiments. Significant differences were determined by t-test or one-way ANOVA with SPSS 13.0 software (SPSS Inc., Chicago, IL, USA). *p* ≤ 0.05 was considered statistically significant.
